# Thyroid markers in early to mid-pregnancy and risk of gestational diabetes: a longitudinal retrospective cohort study

**DOI:** 10.3389/fendo.2026.1799406

**Published:** 2026-05-14

**Authors:** Xiaosong Liu, Chang Zou, Qinxin Shen, Yuanyuan Yang, Yang Liu, Ruru Zhao, Xiao Chen, Qiaoling Du

**Affiliations:** Shanghai Key Laboratory of Maternal Fetal Medicine, Shanghai Institute of Maternal-Fetal Medicine and Gynecologic Oncology, Shanghai First Maternity and Infant Hospital, School of Medicine, Tongji University, Shanghai, China

**Keywords:** free thyroxine (FT4), free triiodothyronine (FT3), FT3/FT4 ratio, gestational diabetes, pregnancy, thyroid markers

## Abstract

**Background:**

Thyroid markers have been associated with an increased risk of gestational diabetes mellitus (GDM), but existing evidence remains inconsistent. The purpose of this study was to examine the associations between free T3 (FT3), thyroid autoimmunity, FT3/free T4 (FT4) ratio, and gestational diabetes mellitus (GDM) in a Chinese population.

**Methods:**

This longitudinal cohort study enrolled 10, 258 women at Shanghai First Maternity and Infant Hospital between January 2023 and November 2025, of whom 7, 463 were included in the final analysis. Thyroid function was assessed at 4–24 gestational weeks, and GDM was diagnosed by a 75-g oral glucose tolerance test (OGTT) at 24–28 weeks, with GDM as the primary outcome.

**Results:**

In early (4–12 weeks) and mid-pregnancy (13–24 weeks), FT3 and the FT3/FT4 ratio were both positively associated with GDM. Lipid metabolism differed significantly across percentiles of the FT3/FT4 ratio. Thyroid peroxidase antibody (TPOAb) was associated with GDM only in early pregnancy, whereas thyroglobulin antibody (TGAb), FT4, and TSH showed no significant associations with GDM.

**Conclusion:**

A higher FT3/FT4 ratio in early and mid-pregnancy was associated with abnormal glucose and lipid metabolism and was positively associated with GDM risk, suggesting its potential role as a supplementary marker for early risk stratification of GDM.

## Introduction

Gestational diabetes mellitus (GDM) is the abnormal glucose tolerance first observed during pregnancy (1). Over the past few decades, GDM prevalence continued to rise worldwide (2). The rising trend was considered attributable to epidemiological factors, including increasing prevalence of type 2 diabetes (T2D), higher proportion of obesity in women of childbearing age, and advanced conception age (3, 4). To date, GDM has imposed a substantial burden on public health worldwide. It exerts adverse effects on maternal and infant health, leading to higher risks of maternal complications (pre-eclampsia, gestational hypertension, postpartum T2D, and cardiovascular disease) (5, 6), as well as neonatal adverse outcomes, including macrosomia, shoulder dystocia, respiratory distress, and neonatal hypoglycemia (7). Recent epidemiological evidence also indicates higher risks of adverse cardiometabolic outcomes and glucose metabolism disturbance later in life of the offspring in GDM cases (8).

Thyroid hormones (THs) play a critical role in energy metabolism by enhancing pancreatic islet cell function and proliferation, as well as regulating glucose homeostasis (9). Several studies investigated the potential connection between thyroid function and the development of GDM, which predominantly focused on the abnormal variations in free thyroxine (FT4) and thyroid-stimulating hormone (TSH) levels (10–12); however, relatively few have examined the role of free triiodothyronine (FT3) (10). Moreover, findings regarding the relationship between maternal thyroid function and glucose metabolism disorders remain inconsistent. Some studies reported an increased GDM risk associated with elevated FT3 levels in the first trimester (11), whereas others have observed nonsignificant or even opposing associations (12, 13).

Among THs, Triiodothyronine (T3) plays an essential role in endogenous glucose production. T3 stimulates the proliferation and maturation of pancreatic β-cells, thus regulating insulin secretion and glucose uptake (14). The ratio of FT3 to FT4 is commonly regarded as a surrogate measure of peripheral deiodinase activity (15). Relative to single TH parameters, composite indices such as the FT3/FT4 ratio may better reflect overall TH homeostasis. Considering the close link between thyroid function and glucose metabolism, investigating the correlation between FT3/FT4 levels and the risk of GDM is of substantial significance. However, existing studies are often limited by single time-point measurements and relatively small sample sizes, and have predominantly focused on early pregnancy (16, 17).

Considering that lipid parameters have been implicated in insulin resistance and the development of GDM, this retrospective cohort study, based on a large-scale Chinese population, primarily aimed to evaluate the associations between thyroid function—particularly the FT3/FT4 ratio—during early and mid-pregnancy and the risk of GDM. We hypothesized that higher FT3 levels and an elevated FT3/FT4 ratio during early and mid-pregnancy are associated with an increased risk of GDM and adverse glucose and lipid metabolic profiles. In addition, we conducted exploratory analyses to examine the relationships between thyroid markers and glucose and lipid metabolism, thereby facilitating the early identification of GDM risk.

## Materials and methods

### Study design and study population

**Figure 1:**
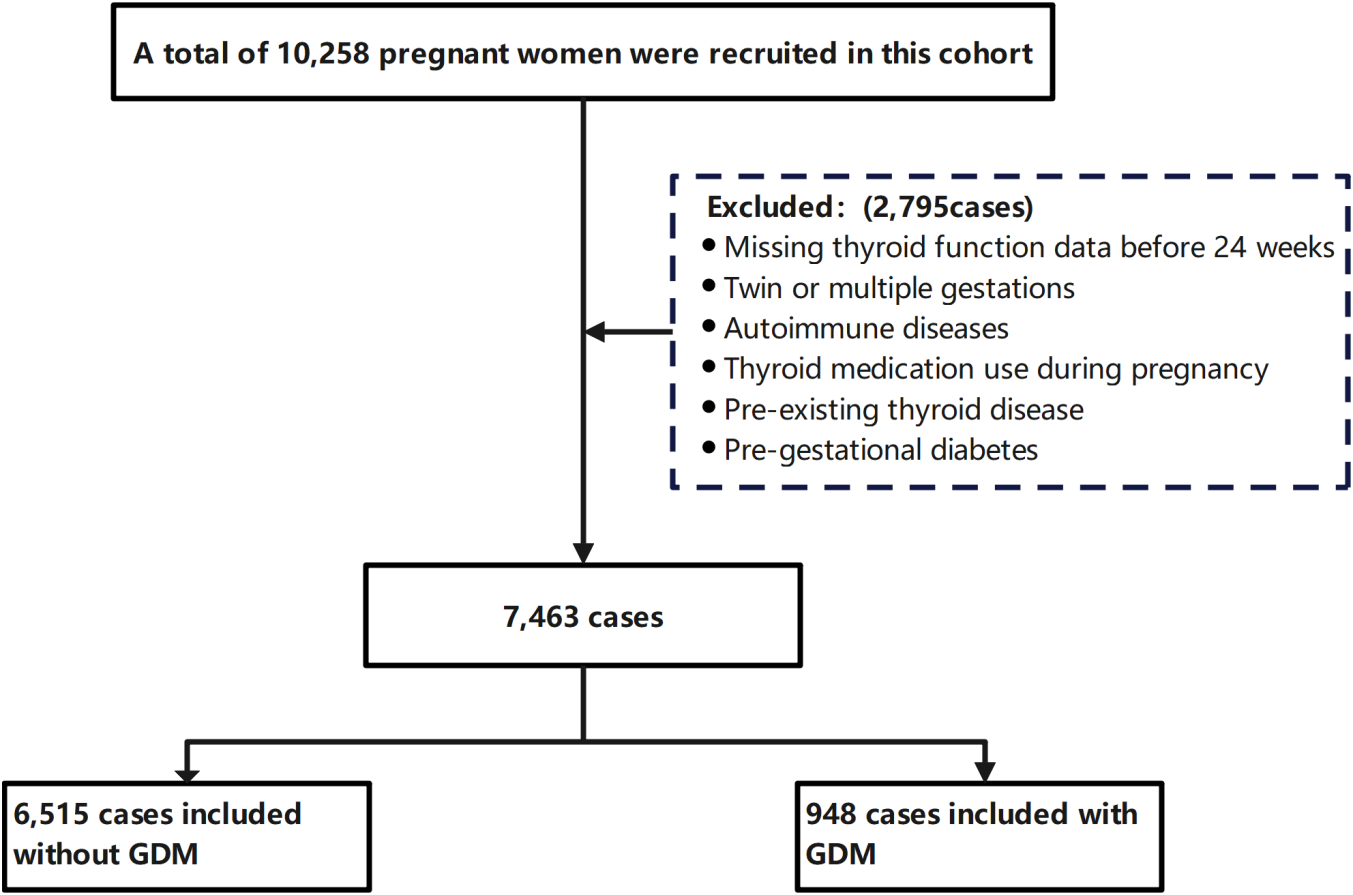
Flow chart of the protocol used to select the study population. GDM, Gestational Diabetes Mellitus.

This was a hospital-based retrospective cohort study conducted at the Shanghai First Maternity and Infant Hospital. We analyzed electronic medical records of 10, 258 pregnant women who received prenatal care and delivered at this hospital between January 2023 and November 2025. Women were excluded if they met any of the following criteria: (1) missing thyroid function test data before 24 weeks of gestation; (2) twin or multiple gestations; (3) presence of autoimmune diseases; (4) thyroid medication use during pregnancy; (5) pre-existing thyroid disease; (6) pre-gestational diabetes mellitus. Consequently, 2, 795 women were excluded, and 7, 463 cases were enrolled in the analysis. Among the included participants, 948 were diagnosed with GDM, and 6, 515 were taken as controls (**Figure 1**). The study was approved by the Ethics Committee of the Shanghai First Maternity and Infant Hospital, School of Medicine, Tongji University (Approval Number: KS1998) and was reported in accordance with the Strengthening the Reporting of Observational Studies in Epidemiology (STROBE) guidelines for cohort studies.

### Laboratory assessment procedures

Maternal peripheral blood samples were collected at the first antenatal visit and centrifuged at 3, 000 rpm for 10 minutes to obtain serum. Serum levels of FT3(Siemens Cat. # 10995584, RRID: AB_3675939; http://antibodyregistry.org/AB_3675939), FT4(Siemens Cat. # 10995588, RRID: AB_2895179; http://antibodyregistry.org/AB_2895179), TSH(Siemens Cat. # 10995704, RRID: AB_2895183, http://antibodyregistry.org/AB_2895183), thyroglobulin antibody (TGAb)(Siemens Cat. # 11201761, RRID: AB_3675829; http://antibodyregistry.org/AB_3675829), and thyroid peroxidase antibody (TPOAb)(Siemens Cat. # 10995467, RRID: AB_3675581; http://antibodyregistry.org/AB_3675581)were measured by the hospital clinical laboratory using the ADVIA Centaur fully automated analyzer, with commercial fluorescence and chemiluminescence immunoassay kits (Siemens, Munich, Germany) according to the manufacturer’s instructions. Elevated TPOAb and TGAb levels were defined as ≥ 60 U/mL, based on the manufacturer-recommended cutoff values. Serum ferritin was quantified using Beckman Coulter kits on UniCel DxI 800 analyzers. Low-density lipoprotein cholesterol (LDL-C) and high-density lipoprotein cholesterol (HDL-C) levels were determined using a homogeneous phase assay, and total cholesterol (TC) and triglyceride (TG) levels were measured by an enzymatic method, using a Beckman Coulter automatic biochemical analyzer (Beckman Coulter Experimental System, Suzhou, China). Early pregnancy was defined as the 4^th^ to 12^th^ weeks of gestation, and mid-pregnancy was defined as 13^th^ to 24^th^ week of gestation.

### Diagnosis of gestational diabetes mellitus

All participants underwent a 75-g oral glucose tolerance test (OGTT) between 24 and 28 weeks of gestation. The diagnosis of GDM was based on the American Diabetes Association (ADA) guidelines (18). GDM was diagnosed if any one of the blood glucose values was at or above the following thresholds in the routine 75 g oral glucose tolerance test (OGTT) at 24–28 weeks of gestation: fasting 5.1 mmol/L, 1-hour 10.0 mmol/L and 2-hour 8.5 mmol/L.

### Covariates and clinical data collection

Following variables were collected by retrospective chart review: age, parity (nulliparous or parous), pre-pregnancy weight, education level, employment status, height, family history of diabetes, history of GDM, mode of delivery (vaginal delivery, assisted vaginal delivery, or cesarean section), gestational age at delivery, newborn sex (male or female), birth weight, 1-minute and 5-minute Apgar scores (divided in 0–3, 4–7, or 8–10), and diagnoses of pregnancy complications.

Body mass index (BMI) was calculated as weight (kg) divided by the square of height (m²) and categorized as underweight (BMI < 18.5 kg/m²), normal weight (BMI 18.5 to < 24 kg/m²), overweight (BMI 24 to < 28 kg/m²), or obese (BMI ≥ 28 kg/m²). Potential sources of bias were considered in this study. Selection bias may exist due to the single-center retrospective design and the exclusion of participants without complete thyroid function data, which may limit generalizability. Information bias may also be present due to the retrospective nature of clinical data collection and the measurement of thyroid function at different gestational time points within predefined intervals. In addition, although multivariable models were adjusted for major confounders, residual confounding from unmeasured factors such as dietary iodine intake and lifestyle factors cannot be excluded.

### Statistical analyses

Descriptive statistics were conducted for demographic information. The normality of continuous variables was assessed prior to analysis. Variables with an approximately normal distribution were presented as mean ± standard deviation (SD), whereas skewed variables were expressed as median with interquartile range (IQR; 25th–75th percentiles). Categorical variables were summarized as numbers (percentages). Between-group comparisons were conducted using the Student’s t test for normally distributed continuous variables and the Mann–Whitney U test for non-normally distributed variables. Categorical variables were compared using the chi-square test when the expected cell counts were ≥5, or Fisher’s exact test otherwise. To evaluate whether glucose and lipid parameters increased progressively across FT3/FT4 ratio quartiles, and to assess trends in the risk of GDM with increasing FT3/FT4 quartiles, P values for linear trend were calculated. Tests of linear trend were performed by modeling the median of each quartile as a continuous variable in the regression models: linear regression was used for continuous outcomes, and logistic regression was applied for binary outcomes. To further explore potential nonlinear associations between serum FT3 levels, the FT3/FT4 ratio, and GDM risk, restricted cubic spline (RCS) regression analyses were performed using logistic regression models. Nonlinearity was assessed by testing the significance of spline terms. To control for potential confounding, multivariable logistic regression models were constructed with adjustment for maternal age, BMI, history of GDM, education level, employment status (employed/unemployed), and family history of diabetes. Statistical analyses were conducted using R statistical software version 4.5.1 (packages “rms”, “dplyr”, “tibble”, and “ggplot2”). All tests were two-sided, and P<0.05 was considered statistically significant.

## Results

### Participant characteristics

A total of 7, 463 women were ultimately included in our study (the flowchart in **Figure 1**). Among them, 948 (12.7%) were diagnosed with GDM. The remaining 6, 515 women were classified into the non-GDM group. **Table 1** summarizes the baseline characteristics of all the participants according to GDM status. Women in the GDM group showed advanced conception age, higher BMI, slightly shorter gestational age at delivery, a higher proportion of multiparous pregnancies, a higher rate with a family history of diabetes, and higher lipid levels (all P<0.05). Ferritin levels were comparable between the two groups (P > 0.05) and were therefore not included in further analyses. As for the distributions of obstetric outcomes, GDM exhibited a significantly higher cesarean delivery rate and elevated risks of pregnancy-induced hypertension, preeclampsia, and macrosomia (all P<0.05).

**Table 1:** Baseline characteristics, perinatal outcomes, and neonatal characteristics between women with and without GDM.

Variables	Overall N = 7, 463	Non-GDM N = 6, 515	GDM N = 948	P-value
Age (years)	31.8 ± 3.9	31.6 ± 3.9	33.1 ± 4.1	<0.001
pre-pregnancy BMI	21.8 ± 3.3	21.6 ± 3.2	23.0 ± 3.7	<0.001
< 18.5kg/m2	898 (12.0%)	836 (12.8%)	62 (6.5%)	
18.5kg/m2-<24kg/m2	4, 740 (63.5%)	4, 197 (64.4%)	543 (57.3%)	
24kg/m2-<28kg/m2	1, 084 (14.5%)	881 (13.5%)	203 (21.4%)	
>=28kg/m2	378 (5.1%)	285 (4.4%)	93 (9.8%)	
Education Level				0.055
Bachelor’s Degree	3, 968 (53.2%)	3, 456 (53.0%)	512 (54.0%)	
Master’s/Doctoral Degree	1, 719 (23.0%)	1, 531 (23.5%)	188 (19.8%)	
Associate Degree	995 (13.3%)	858 (13.2%)	137 (14.5%)	
High School or Less	781 (10.5%)	670 (10.3%)	111 (11.7%)	
Employment status (unemployed %)	243 (3.3%)	212 (3.3%)	31 (3.3%)	>0.9
Parity				<0.001
0	5, 673 (76.0%)	5, 011 (76.9%)	662 (69.8%)	
1	1, 525 (20.4%)	1, 284 (19.7%)	241 (25.4%)	
2	241 (3.2%)	202 (3.1%)	39 (4.1%)	
≥3	24 (0.3%)	18 (0.3%)	6 (0.6%)	
History of gestational diabetes mellitus (%)	4 (0.1%)	1 (0.0%)	3 (0.3%)	0.003
Family history of diabetes	148 (2.0%)	110 (1.7%)	38 (4.0%)	<0.001
Insulin (uU/mL)	7.0 (5.0, 10.1)	6.8 (4.9, 9.6)	9.0 (6.1, 13.5)	<0.001
0 h glucose OGTT (mmol/L)	4.2 (4.0, 4.4)	4.1 (4.0, 4.3)	4.5 (4.2, 4.8)	<0.001
1 h glucose OGTT (mmol/L)	7.5 (6.4, 8.6)	7.2 (6.3, 8.1)	10.1 (9.1, 10.7)	<0.001
2 h glucose OGTT (mmol/L)	6.4 (5.6, 7.3)	6.2 (5.5, 6.9)	8.7 (8.0, 9.5)	<0.001
Systolic blood pressure (mmHg)	121.4 ± 13.3	121.3 ± 13.2	122.0 ± 14.0	0.14
Diastolic blood pressure (mmHg)	78.2 ± 9.1	78.1 ± 9.1	79.0 ± 9.2	0.003
Fasting TC (mmol/L)	4.6 (4.1, 5.1)	4.6 (4.1, 5.1)	4.7 (4.2, 5.2)	0.004
Fasting TG (mmol/L)	1.3 (1.0, 1.7)	1.2 (1.0, 1.6)	1.4 (1.1, 2.0)	<0.001
Fasting HDL-C (mmol/L)	1.9 (1.7, 2.2)	2.0 (1.7, 2.2)	1.9 (1.6, 2.2)	<0.001
Fasting LDL-C (mmol/L)	2.6 (2.2, 3.1)	2.6 (2.2, 3.0)	2.7 (2.2, 3.1)	<0.001
ALT(U/L)	13.0 (10.0, 20.0)	13.0 (10.0, 19.0)	15.0 (10.0, 23.0)	<0.001
TBil(umol/L)	2.4 ± 2.5	2.4 ± 2.5	2.6 ± 2.1	0.088
Ferritin (ng/mL)	43.8 (24.1, 74.5)	43.8 (24.0, 74.7)	43.7 (25.7, 74.1)	0.5
Delivery mode				0.011
Cesarean Section	3, 946 (52.9%)	3, 413 (52.4%)	533 (56.2%)	
Vaginal Delivery	3, 220 (43.1%)	2, 844 (43.7%)	376 (39.7%)	
Assisted Vaginal Delivery	150 (2.0%)	137 (2.1%)	13 (1.4%)	
Perinatal outcomes				
PIH (%)	645 (8.6%)	531 (8.2%)	114 (12.0%)	<0.001
PE (%)	314 (4.2%)	257 (3.9%)	57 (6.0%)	0.003
ICP (%)	147 (2.0%)	132 (2.0%)	15 (1.6%)	0.4
PROM (%)	1, 786 (23.9%)	1, 549 (23.8%)	237 (25.0%)	0.4
Neonatal outcomes				
Birth age (weeks)	38.5 (38.0, 39.3)	38.6 (38.0, 39.3)	38.4 (37.4, 39.1)	<0.001
Birth weight (g)	3, 170.0 (2, 870.0, 3, 445.0)	3, 165.0 (2, 875.0, 3, 435.0)	3, 185.0 (2, 835.0, 3, 500.0)	0.081
Macrosomia	189 (2.5%)	145 (2.2%)	44 (4.6%)	<0.001
Apgar score at 1 minute				0.7
0~3	49 (0.7%)	44 (0.7%)	5 (0.5%)	
4~7	71 (1.0%)	64 (1.0%)	7 (0.7%)	
8~10	7, 342 (98.4%)	6, 406 (98.3%)	936 (98.7%)	
Apgar score at 5 minutes				0.6
0~3	42 (0.6%)	37 (0.6%)	5 (0.5%)	
4~7	11 (0.1%)	11 (0.2%)	0 (0.0%)	
8~10	7, 409 (99.3%)	6, 466 (99.2%)	943 (99.5%)	

P<0.05 represents a significant difference between the two groups.

BMI, body mass index; OGTT, oral glucose tolerance test; TC, total cholesterol; TG, triglyceride; HDL, high-density lipoprotein cholesterol; LDL, low-density lipoprotein cholesterol; ALT, alanine aminotransferase; TBil, total bilirubin; PIH, pregnancy-induced hypertension; PE, preeclampsia; ICP, intrahepatic cholestasis of pregnancy; PROM, premature rupture of membranes.

### Thyroid hormone profiles of gestational diabetes mellitus cases

**Table 2** illustrates the distributions of TH parameters stratified by GDM status across different gestational periods. There were significant differences in thyroid function (according to the levels of FT3, FT4, and the FT3/FT4 ratio) observed in GDM cases compared to the control pregnancies at different stages of pregnancy. GDM cases showed significant variations in FT3 and FT4 levels of early and mid-pregnancy. Specifically, compared with the non-GDM group, women with GDM exhibited higher FT3 levels (p < 0.05) and FT3/FT4 ratios (p < 0.001), along with lower FT4 levels (p < 0.05). In addition, the prevalence of TPOAb positivity during early pregnancy was significantly higher in the GDM group than in the non-GDM group (p < 0.001), whereas no significant differences in TGAb positivity were observed between the two groups at any gestational stage.

**Table 2:** Thyroid hormone (TH) levels in women with and without GDM.

Variables	Overall	Non-GDM	GDM	P-value
4~24 weeks	N = 7463	N = 6515	N = 948	
FT3 (ng/dL)	4.9 (4.5, 5.2)	4.9 (4.5, 5.2)	4.9 (4.6, 5.3)	<0.001
FT4 (ng/dL)	15.3 (14.1, 16.6)	15.3 (14.1, 16.6)	15.1 (13.9, 16.4)	0.002
TSH (uIU/mL)	1.1 (0.6, 1.8)	1.1 (0.6, 1.8)	1.1 (0.6, 1.7)	0.5
FT3/FT4	0.3 (0.3, 0.3)	0.3 (0.3, 0.3)	0.3 (0.3, 0.4)	<0.001
TPOAB positive	847 (11.3%)	711 (10.9%)	136 (14.3%)	0.010
TGAB positive	215 (3.8%)	190 (3.9%)	25 (3.6%)	0.8
4~12weeks	N=4854	N=4211	N=643	
FT3	4.9 (4.6, 5.3)	4.9 (4.6, 5.3)	5.0 (4.6, 5.4)	0.017
FT4	15.6 (14.4, 16.8)	15.6 (14.4, 16.9)	15.3 (14.2, 16.6)	0.003
TSH	1.1 (0.5, 1.7)	1.1 (0.5, 1.8)	1.0 (0.6, 1.6)	0.4
FT3/FT4	0.3 (0.3, 0.3)	0.3 (0.3, 0.3)	0.3 (0.3, 0.4)	<0.001
TPOAB positive	540 (11.1%)	440 (10.4%)	100 (15.6%)	<0.001
TGAB positive	140 (3.8%)	122 (3.7%)	18 (3.8%)	>0.9
13~24 weeks	N=2609	N=2304	N=305	
FT3	4.7 (4.4, 5.1)	4.7 (4.4, 5.1)	4.8 (4.5, 5.2)	<0.001
FT4	14.8 (13.6, 16.1)	14.8 (13.6, 16.1)	14.6 (13.2, 15.9)	0.021
TSH	1.2 (0.7, 1.9)	1.2 (0.7, 1.9)	1.3 (0.8, 1.8)	>0.9
FT3/FT4	0.3 (0.3, 0.3)	0.3 (0.3, 0.3)	0.3 (0.3, 0.4)	<0.001
TPOAb positive	307 (11.8%)	271 (11.8%)	36 (11.8%)	>0.9
TGAb positive	75 (4.0%)	68 (4.1%)	7 (3.1%)	0.6

FT4, free thyroxine; FT3, free triiodothyronine; TSH, Thyroid Stimulating Hormone; TPOAb, Thyroid peroxidase antibodies; TGAb, thyroglobulin antibody.

### Multivariable-adjusted associations between thyroid hormones and gestational diabetes mellitus risk

**Figure 2:**
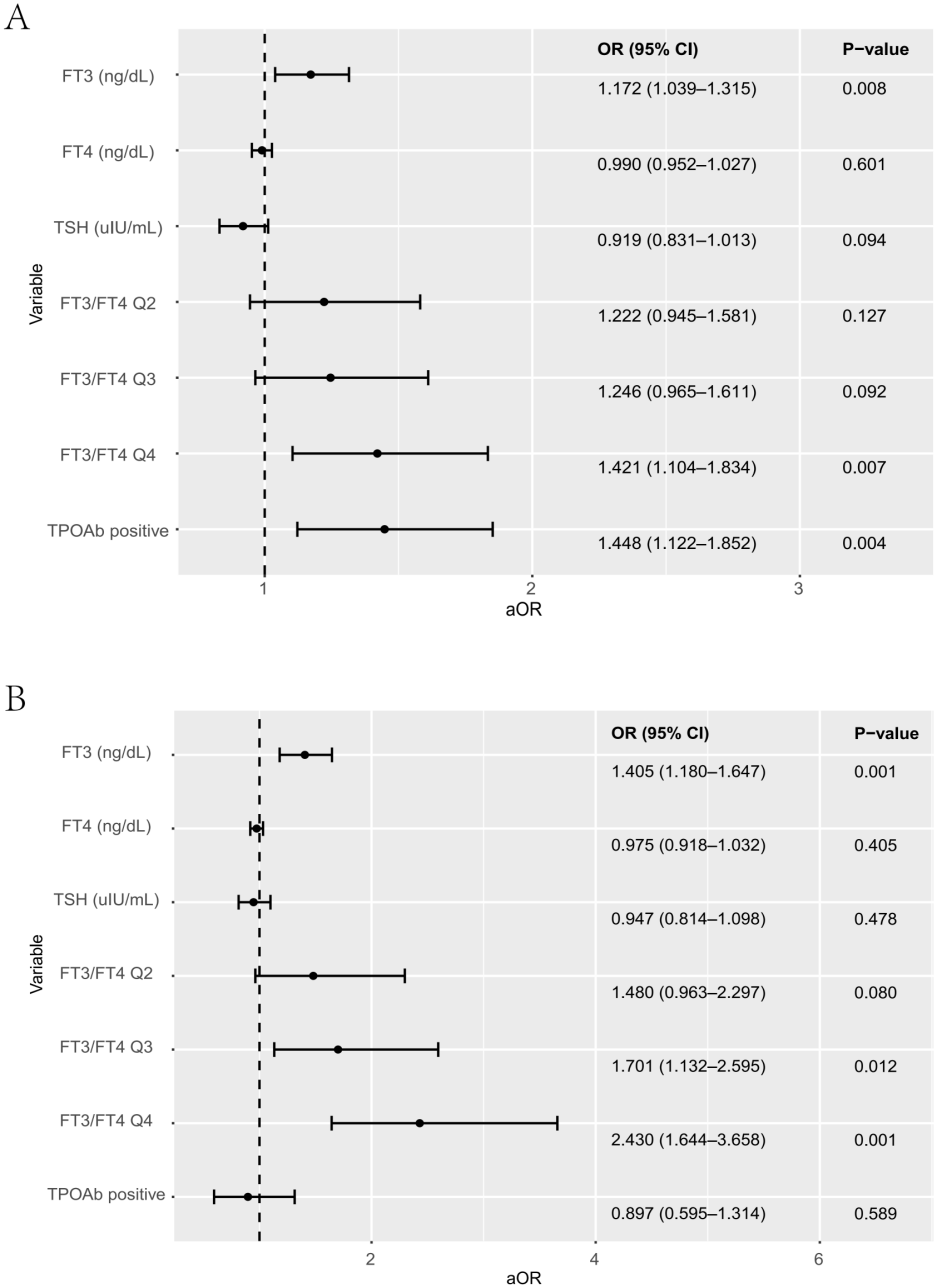
Comparison of thyroid hormone levels between women with GDM and non-GDM women during early pregnancy **(A)** and mid-pregnancy **(B)**. Multivariable linear regression was applied with adjustment for maternal age(years), pre-pregnancy BMI (kg/m²), family history of diabetes(yes or no), education level, employment status (employed/unemployed), and parity(0, 1, 2, or ≥3).

To further evaluate glucose and lipid metabolism and the risk of GDM in relation to the FT3/FT4 ratio during early and mid-pregnancy, participants were divided into quartiles based on the distribution of the FT3/FT4 ratio [quartile 1 (Q1), ≤0.289; quartile 2 (Q2), 0.289–0.315; quartile 3 (Q3), 0.315–0.345; and quartile 4 (Q4), ≥0.345]. **Table 3**, **Figure 2** present the crude and multivariable-adjusted odds ratios (aORs) of GDM according to FT3/FT4 ratio quartiles. After adjusting for conception age, BMI, family history of diabetes mellitus, education level, employment status, and GDM history, FT3 levels were positively associated with the development of GDM both in early pregnancy [aOR 1.172 (95%CI 1.039-1.315), p = 0.008] and mid-pregnancy [aOR 1.405 (95%CI 1.180-1.647), p < 0.001]. Consistently, a significantly higher risk of GDM was observed in women within the highest quartile (Q4) of the FT3/FT4 ratio during early pregnancy compared with those in the lowest quartile (Q1) [aOR 1.421 [95%CI 1.104-1.834), p = 0.007]. During mid-pregnancy, higher FT3/FT4 ratios were consistently associated with an increased risk of GDM. Specifically, women with higher FT3/FT4 ratios (Q3 and Q4) had significantly higher risks of GDM compared with those in Q1 [Q3, aOR 1.701 (95%CI 1.132-2.595), p = 0.012; Q4, aOR 2.430 (95%CI 1.644-3.658), p < 0.001]. Meanwhile, TPOAb positivity was associated with an increased risk of GDM only in early pregnancy. Neither FT4 nor TSH levels were significantly associated with GDM risk at any gestational stage. Overall, these findings suggest a strong association between maternal TH profiles, particularly the FT3/FT4 ratio, and the risk of GDM throughout pregnancy.

**Table 3:** Comparison of TH levels between GDM and non-GDM women during early to mid-pregnancy.

Variables	OR (95% CI)	P	aOR (95% CI)	P-value
4~12weeks				
FT3(ng/dL)	1.144 [1.021-1.276]	0.018	1.172 [1.039-1.315]	0.008
FT4(ng/dL)	0.955 [0.918-0.991]	0.017	0.990 [0.952-1.027]	0.601
TSH(uIU/mL)	0.951 [0.865-1.043]	0.288	0.919 [0.831-1.013]	0.094
FT3/FT4 Q2	1.272 [0.995-1.629]	0.056	1.222 [0.945-1.581]	0.127
FT3/FT4 Q3	1.394 [1.091-1.785]	0.008	1.246 [0.965-1.611]	0.092
FT3/FT4 Q4	1.693 [1.334-2.155]	<0.001	1.421 [1.104-1.834]	0.007
TPOAB positive	1.575 [1.240-1.985]	<0.001	1.448 [1.122-1.852]	0.004
13~24 weeks				
FT3(ng/dL)	1.268 [1.091-1.472]	0.002	1.405 [1.180-1.674]	<0.001
FT4(ng/dL)	0.932 [0.880-0.984]	0.014	0.975 [0.918-1.032]	0.405
TSH(uIU/mL)	0.969 [0.843-1.109]	0.65	0.947 [0.814-1.098]	0.478
FT3/FT4 Q2	1.578 [1.050-2.396]	0.030	1.480 [0.963-2.297]	0.080
FT3/FT4 Q3	1.980 [1.348-2.951]	<0.001	1.701 [1.132-2.595]	0.012
FT3/FT4 Q4	2.839 [1.970-4.166]	<0.001	2.430 [1.644-3.658]	<0.001
TPOAB positive	1.006 [0.685-1.440]	0.974	0.897 [0.595-1.314]	0.589

The FT3/FT4 ratio was categorized into quartiles based on its distribution among pregnant women.

aOR, adjusted Odds Ratio: Multivariable linear regression was applied with adjustment for maternal age (years), pre-pregnancy BMI (kg/m²), family history of diabetes (yes or no), education level, employment status (employed/unemployed), and parity (0, 1, 2, or ≥3).

### Glucose and lipid metabolic profiles across FT3/FT4 ratio quartiles

During both early and mid-pregnancy, statistically significant linear trends were observed across increasing FT3/FT4 ratio quartiles for TC, TG, HDL-C, LDL-C, fasting insulin levels, fasting glucose, 1-hour glucose, and 2-hour glucose during the OGTT, as well as for the incidence of GDM (all P for linear trend < 0.05). The highest fasting insulin concentrations, OGTT glucose values, and the highest GDM incidence were observed in the third FT3/FT4 ratio quartile in both gestational periods, whereas the highest lipid levels were observed in the first and fourth quartiles. Detailed results were shown in **Table 4**.

**Table 4:** Glucose and lipid metabolism according to FT3/FT4 ratio quartiles.

Variables	Quartile 1	Quartile 2	Quartile 3	Quartile 4	P for linear trend
4~12 weeks	N=1214	N=1214	N=1212	N=1214	
FT3/FT4 ratio range	≤0.289	0.289-0.315	0.315-0.345	≥0.345	
0 h glucose OGTT (mmol/L)	4.10 [3.90, 4.30]	4.10 [4.00, 4.40]	4.20 [4.00, 4.40]	4.20 [4.00, 4.50]	<0.001
1 h glucose OGTT (mmol/L)	7.40 [6.30, 8.50]	7.40 [6.40, 8.60]	7.50 [6.50, 8.60]	7.70 [6.60, 8.70]	<0.001
2 h glucose OGTT (mmol/L)	6.20 [5.50, 7.10]	6.40 [5.60, 7.30]	6.40 [5.60, 7.30]	6.60 [5.80, 7.60]	<0.001
GDM	126 (10%)	151 (12%)	168 (14%)	198 (16%)	0.006
TC (mmol/L)	4.55 [4.13, 5.06]	4.52 [4.07, 4.97]	4.51 [4.06, 4.98]	4.48 [3.99, 5.00]	<0.001
TG (mmol/L)	1.13 [0.88, 1.48]	1.16 [0.90, 1.53]	1.21 [0.95, 1.57]	1.28 [0.96, 1.70]	<0.001
HDL-C (mmol/L)	2.00 [1.70, 2.27]	1.95 [1.70, 2.21]	1.92 [1.67, 2.16]	1.82 [1.55, 2.09]	<0.001
LDL-C (mmol/L)	2.50 [2.12, 2.96]	2.49 [2.09, 2.93]	2.56 [2.13, 2.99]	2.59 [2.17, 3.09]	0.648
Iinsulin (uU/mL)	6.3 [4.6, 8.4]	6.6 [4.8, 9.1]	7.3 [5.3, 10.5]	8.4 [5.8, 12.5]	<0.001
13~24 weeks	N=653	N=652	N=653	N=651	
FT3/FT4 ratio range	≤0.292	0.292-0.319	0.319-0.349	≥0.349	
0 h glucose OGTT (mmol/L)	4.10 [3.90, 4.30]	4.10 [3.90, 4.30]	4.20 [4.00, 4.40]	4.20 [4.00, 4.50]	<0.001
1 h glucose OGTT (mmol/L)	7.10 [6.10, 8.10]	7.40 [6.30, 8.40]	7.50 [6.40, 8.60]	7.60 [6.60, 8.70]	<0.001
2 h glucose OGTT (mmol/L)	6.10 [5.40, 6.95]	6.20 [5.40, 7.00]	6.40 [5.50, 7.30]	6.50 [5.70, 7.40]	<0.001
GDM	46 (7.0%)	65 (10.0%)	85 (13.0%)	109 (16.7%)	<0.001
TC (mmol/L)	4.77 [4.31, 5.40]	4.75 [4.28, 5.30]	4.75 [4.22, 5.37]	4.76 [4.28, 5.48]	0.457
TG (mmol/L)	1.35 [1.02, 1.77]	1.31 [1.02, 1.76]	1.40 [1.06, 1.81]	1.57 [1.19, 2.09]	0.002
HDL-C (mmol/L)	2.05 [1.77, 2.34]	2.06 [1.80, 2.35]	1.98 [1.72, 2.25]	1.89 [1.60, 2.16]	<0.001
LDL-C (mmol/L)	2.59 [2.18, 3.08]	2.64 [2.22, 3.11]	2.66 [2.23, 3.23]	2.81 [2.30, 3.33]	0.007
Iinsulin(uU/mL)	5.8 [4.2, 8.2]	6.4 [5.0, 8.5]	7.0 [5.2, 10.3]	8.7 [5.9, 12.2]	<0.001

*Participants were categorized into quartiles based on the FT3/FT4 ratio. Continuous variables are presented as median (interquartile range), and categorical variables as number (percentage). P for linear trend across quartiles was assessed by modeling the median value of each quartile as a continuous variable, using linear regression for continuous outcomes and logistic regression for binary outcomes.

*Multivariable linear regression was applied with adjustment for maternal age(years), pre-pregnancy BMI (kg/m²), family history of diabetes(yes or no), education level, employment status (employed/unemployed), and parity(0, 1, 2, or ≥3).

### Linear associations of FT3 and FT3/FT4 ratio with GDM risk

**Figure 3:**
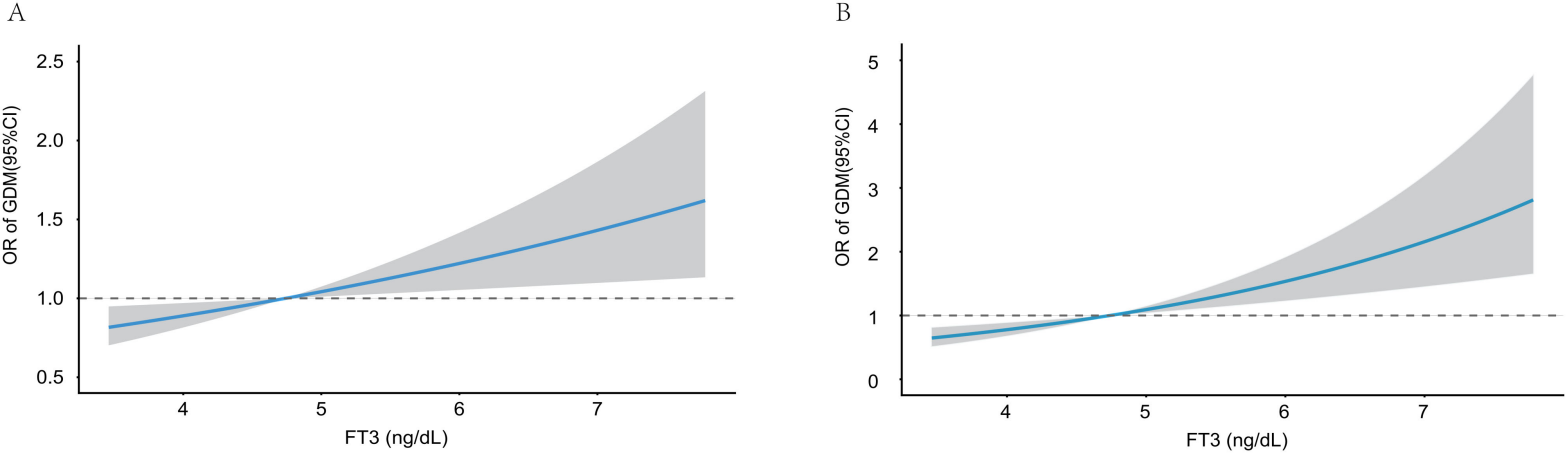
Association of FT3 with the risk of GDM during early **(A)** and mid-pregnancy **(B)**. Multivariable linear regression was applied with adjustment for maternal age(years), pre-pregnancy BMI (kg/m²), family history of diabetes(yes or no), education level, employment status (employed/unemployed), and parity(0, 1, 2, or ≥3).

**Figure 4:**
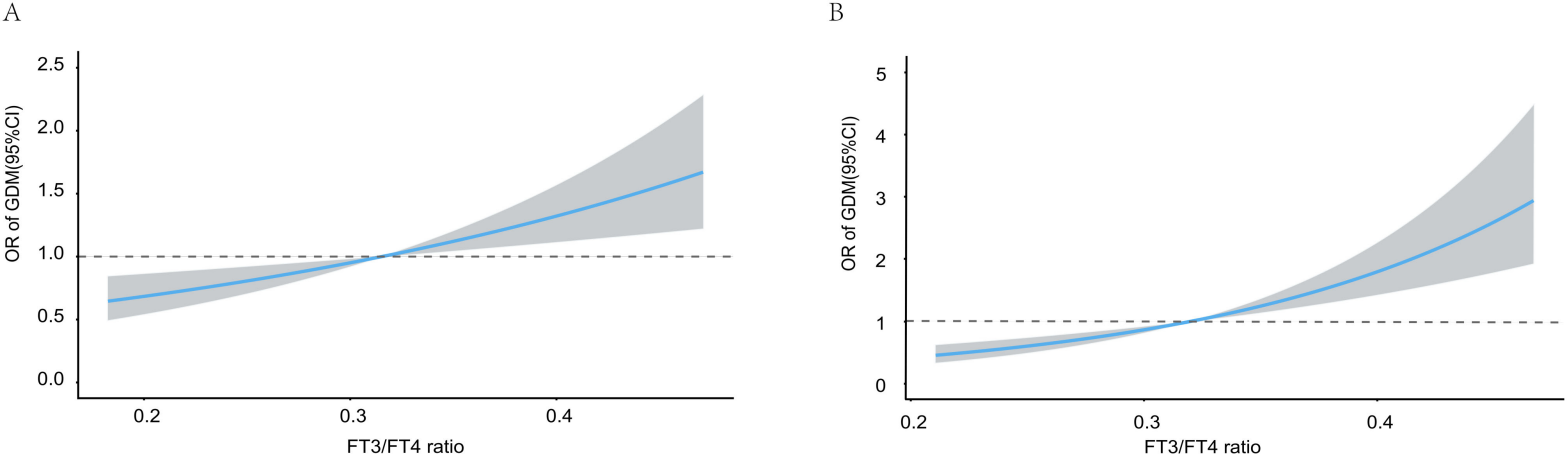
Association of FT3/FT4 ratio with the risk of GDM during early **(A)** and mid-pregnancy **(B)**. Multivariable linear regression was applied with adjustment for maternal age(years), pre-pregnancy BMI (kg/m²), family history of diabetes(yes or no), education level, employment status (employed/unemployed), and parity(0, 1, 2, or ≥3).

Restricted cubic spline (RCS) analyses were first performed to assess potential nonlinear associations of serum FT3 levels and the FT3/FT4 ratio with the risk of GDM. No significant evidence of nonlinearity was detected for either FT3 or the FT3/FT4 ratio during early or mid-pregnancy. Therefore, subsequent analyses focused on linear associations. As shown in **Figure 3**, higher serum FT3 levels were linearly associated with an increased risk of GDM during both early pregnancy (**Figure 3A**) and mid-pregnancy (**Figure 3B**). Similarly, a clear positive linear association was observed between the FT3/FT4 ratio and the odds of GDM in both early pregnancy (**Figure 4A**) and mid-pregnancy (**Figure 4B**), with progressively increasing risks at higher FT3/FT4 ratio levels.

## Discussion

Existing data are conflicting on the association between GDM and THs during pregnancy. In this large-scale retrospective cohort study, we observed several findings. Consistent with previous reports, women developing GDM were older at conception, had higher pre-pregnancy BMI, and were more likely to have a family history of diabetes compared with women without GDM. They also exhibited higher lipid levels and a greater risk of delivering macrosomic infants. Further analyses demonstrated that, even after adjusting for potential confounders including maternal age, pre-pregnancy BMI, family history of diabetes, education level, employment status, and history of GDM, serum FT3 levels and the FT3/FT4 ratio during both early and mid-pregnancy remained positively associated with subsequent GDM risk, with the FT3/FT4 ratio showing a stronger association. Taken together, these findings suggest that the FT3/FT4 ratio, as a surrogate marker of peripheral deiodinase activity and TH conversion, may serve as acomplementary indicator of GDM risk than individual TH measurements, in both early and mid-pregnancy.

In this study, the prevalence of GDM was 12.7%, comparable to that reported in a previous study (19). Growing evidence in recent years has suggested a complex interplay between THs and GDM. Multiple mechanisms have been proposed to explain the TH-mediated regulation of glucose metabolism. First, THs regulate liver enzymes involved in gluconeogenesis and glycogenolysis, thereby increasing endogenous glucose production (20, 21). Second, THs enhance expression of the hepatic glucose transporter GLUT2, leading to increased hepatic glucose output (22). Third, THs modulate glucose homeostasis by regulating the differentiation and functional activity of pancreatic islet cells in the maternal pancreas (23). Collectively, THs can influence glucose metabolism during pregnancy in multiple ways. However, existing evidence regarding the effects of maternal THs on gestational glucose metabolism remains inconsistent (24), and the underlying mechanisms, as well as their clinical implications, require further investigation.

Previous studies investigating the association between FT3 and GDM have several limitations. Most studies measured TH levels at a single time point, which may not adequately capture the dynamic changes in thyroid function throughout pregnancy (13, 25). Moreover, some investigations focused exclusively on the independent effects of FT3 or FT4 without fully accounting for the role of peripheral TH conversion in glucose metabolism (10, 25, 26), which may partly explain the inconsistent findings reported to date. Accumulating evidence suggests that FT3 is a biologically active TH primarily responsible for regulating glucose metabolic activity (27–29), and approximately 80% of circulating FT3 is generated through peripheral deiodinase-mediated monodeiodination of FT4 (25, 30). Accordingly, the FT3/FT4 ratio has been proposed as a surrogate marker of type 1 and type 2 deiodinase activity, which governs the peripheral conversion of FT4 to metabolically active FT3, thereby supporting the potential utility of the FT3/FT4 ratio as an indicator of glucose homeostasis (17). In the present study, we characterized longitudinal trajectories of THs before 24 weeks of gestation and observed that FT3 levels during both the first and second trimesters were significantly and positively associated with the risk of GDM, consistent with the findings reported by Ortega et al. (27). Notably, we further identified the FT3/FT4 ratio may represent a potential risk indicator for GDM. The FT3/FT4 ratio, reflecting the efficiency of peripheral T4-to-T3 conversion, showed relatively stronger associations with GDM risk; women with the highest quartile of the FT3/FT4 ratio during early pregnancy had a 42.7% increased risk of GDM, while those in the third and fourth quartiles during mid-pregnancy had a 72.4% and 145.3% higher risk, respectively. Overall, our findings are largely consistent with previous studies (31, 32). Notably, in our study, higher FT3/FT4 ratios were accompanied by elevated maternal lipid levels, which may partially explain the observed associations. Taken together, elevated FT3 levels, which potentially reflect increased *de novo* synthesis or enhanced deiodinase activity, may be involved in the pathophysiology of GDM, although causality cannot be established. Moreover, previous studies examining the association between thyroid function biomarkers and GDM risk have primarily focused on FT4 and TSH (33, 34), whereas evidence regarding FT3 or composite indices remains insufficient. Zhao W et al. reported that the FT4/TSH ratio in early pregnancy was closely associated with glucose and lipid metabolism as well as insulin resistance (35), while no independent effects of FT4 or TSH on GDM risk were observed. Other studies have similarly reported no significant associations between individual FT4 or TSH levels and the risk of GDM (13, 36), a pattern consistent with our findings. In the present study, women with GDM generally exhibit lower FT4 levels and higher TSH levels; however, after adjustment for potential confounders, neither FT4 nor TSH was significantly associated with GDM risk in early or mid-pregnancy. These findings suggest that, within multivariable models, traditional thyroid function parameters show unstable associations with GDM risk, highlighting the limitations of relying solely on single TH measurements.

The association between thyroid autoantibodies and the risk of GDM remains controversial. Previous studies linked TPOAb positivity to several adverse pregnancy outcomes, such as preterm birth and miscarriage (37); however, its relationship with glucose metabolism remained inconsistent. While some studies reported no significant association between thyroid autoimmunity and GDM (38), others observed a significant relation between TPOAb positivity and GDM risk in early pregnancy (39). Our findings are in line with the latter observations. From a mechanistic perspective, thyroid autoantibodies may be associated with subtle impairments in thyroid function or may reflect broader immune activation, particularly immune dysregulation at the maternal-fetal interface (40). In addition, thyroid autoantibodies may compromise follicular epithelial cell integrity, thereby impairing TH synthesis and secretion (41), disrupting glucose uptake and utilization, and ultimately contributing to metabolic dysregulation.

Previous studies have shown that lipid levels increase physiologically during pregnancy and are associated with insulin resistance, thereby increasing the risk of GDM (16). In this study, women with GDM exhibited higher levels of TC, TG, and LDL, and lower HDL levels, consistent with previous findings (42). Further analyses showed that the FT3/FT4 ratio was positively associated with TC, TG, and LDL, and negatively associated with HDL, with similar patterns observed at 13–24 weeks of gestation. These findings suggest that thyroid function may influence GDM risk by modulating lipid metabolism. An elevated FT3/FT4 ratio may reflect increased deiodinase activity, contributing to lipid disturbances and insulin resistance (43, 44). However, the relationship between thyroid function and lipid metabolism remains inconsistent across studies. Overall, our findings provide insight into a potential metabolic pathway linking the FT3/FT4 ratio to GDM, although further studies are needed to confirm these results.

### Strengths and limitations

This study has notable strengths. We longitudinally assessed thyroid function markers, lipid profiles, and glucose tolerance indices from 4 to 24 weeks of gestation, enabling a comprehensive evaluation of the associations between dynamic changes in TH status and GDM risk. Notably, our analyses focused on composite TH indices, which showed more stable associations with GDM. To our knowledge, it represents the largest study to date examining composite TH markers in relation to GDM. In addition, thyroid autoantibodies (TPOAb and TGAb) were measured and accounted for, enabling a more complete assessment of the role of thyroid autoimmunity. The study was conducted in Shanghai, an iodine-sufficient coastal region in China, thereby minimizing potential confounding by iodine deficiency (45). Several limitations should also be acknowledged. Individual iodine level was not measured for confounder adjustment. In addition, the single-center design may limit the generalizability of the findings to other populations, and external validation in multicenter cohorts is warranted. Information on family history of thyroid disease was not collected, which may introduce residual confounding. Finally, the retrospective nature of the study may introduce selection bias and residual confounding from unmeasured factors such as diet, smoking, and alcohol consumption. Prospective studies in diverse populations are therefore warranted to confirm and extend these findings.

## Conclusion

In summary, this large retrospective cohort study found that FT3 levels and the FT3/FT4 ratios in early and mid-pregnancy are positively associated with the risk of GDM. Our findings contribute to the understanding of maternal thyroid-related metabolic alterations during pregnancy and suggest a potential role of thyroid-related markers in GDM risk stratification. Further prospective and mechanistic studies are warranted to validate these findings.

## Data Availability

The raw data supporting the conclusions of this article will be made available by the authors, without undue reservation.
